# Effects of Texture and Grain Size on the Yield Strength of ZK61 Alloy Rods Processed by Cyclic Extrusion and Compression

**DOI:** 10.3390/ma10111234

**Published:** 2017-10-26

**Authors:** Lixin Zhang, Wencong Zhang, Biao Cao, Wenzhen Chen, Junpeng Duan, Guorong Cui

**Affiliations:** 1 School of Materials Science and Engineering, Harbin Institute of Technology at Weihai, Weihai 264209, China; zhanglixn2004@126.com (L.Z.); junpeng.duan@wfjt.com (J.D.); cuiguorong2010@126.com (G.C.); 2 Department of Fundamental Experiment, Naval Aeronautical University, Yantai 264001, China; Caobiao2001@126.com; 3 Weihai Wangfeng Magnesium Industry Science and Technology Develop Co. Ltd., Weihai 264209, China

**Keywords:** ZK61 alloy, cyclic extrusion and compression, crystallographic texture, room-temperature yield strength, extrusion direction

## Abstract

The ZK61 alloy rods with different grain sizes and crystallographic texture were successfully fabricated by cyclic extrusion and compression (CEC). Their room-temperature tension & compression yield strength displayed a significant dependence on grain size and texture, essentially attributed to {10-12} twinning. The texture variations were characterized by the angle *θ* between the c-axis of the grain and the extrusion direction (ED) during the process. The contour map of room-temperature yield strength as a function of grain size and the angle *θ* was obtained. It showed that both the tension yield strength and the compression yield strength of ZK61 alloy were fully consistent with the Hall-Patch relationship at a certain texture, but the change trends of the tension yield strength and the compression yield strength were completely opposite at the same grain size while texture altered. The friction stresses of different deformation modes calculated based on the texture confirmed the tension yield strength of the CECed ZK61 alloy rods, which was determined by both the basal slip and the tension twinning slip during the tension deformation at room temperature, while the compression yield strength was mainly determined by the basal slip during the compression deformation.

## 1. Introduction

In recent years, the ZK61 alloy has attracted more and more attention due to its high strength at room temperature (RT) as a kind of widely used commercial wrought magnesium alloy. Most of the existing work has focused on the effects of texture and grain size on the mechanical properties of ZK61 alloy [1,2,3]. It is because of the hexagonal close-packed (hcp) crystallite structure of magnesium and its alloys that the preferred orientations of the grains caused by deformation are very common phenomena in wrought magnesium alloys [4]. The crystalline texture plays an important role in numerous mechanical and physical behaviors in magnesium alloy. For example, in the previous literatures [5,6,7,8,9], the as-extruded ZK61 alloy rods exhibited an intense fiber texture with {0002} basal plane parallel to the extrusion direction (ED), which lead to an obvious tension/compression asymmetry along the ED. Grain refinement is one of most well-known approaches to significantly strengthening the materials following the Hall-Petch relationship: $\sigma_{0.2}=\sigma_{0}+kd^{-1/2}$ (where $\sigma_{0}$ is the frictional stress, and k is the positive slope).

Severe plastic deformation (SPD) is a very effective technique to enhance the mechanical properties of the ZK61 alloy by the refinement of microstructure. But during the SPD not only the grain size can be refined through dynamic recrystallization (DRX), but most of the grains can also rotate to a certain direction by the plastic deformation. Therefore, grain refinement is always accompanied with the texture changing during the SPD. Cyclic extrusion and compression (CEC) is considered as a powerful processing tool for producing bulk ultrafine grained (UFG) materials as SPD techniques [10,11,12,13,14]. However, the tension strength of these CECed alloys shows an unexpected decrease even though grain size was reduced effectively, which is not consistent with the Hall-Petch relationship. Although the previous studies have shown that this inverse Hall-Petch relationship is caused by texture changes in these CECed alloys [15,16,17,18], there is no way to distinguish the strengthening mechanism of individual texture and grain size on the yield strength until now. Therefore, we fabricated the ZK61 alloy rods with different grain sizes and crystallographic texture by CEC and constructed the room-temperature yield strength contours for the tension and compression as a function of grain size and the texture, which made it possible to distinguish the strengthening mechanism of individual texture and grain size on the yield strength. This can provide an available reference to processing and designing high-performance wrought Mg alloy rods that are suitable for industrial fabrication.

## 2. Experimental Procedure

**Figure 1:**
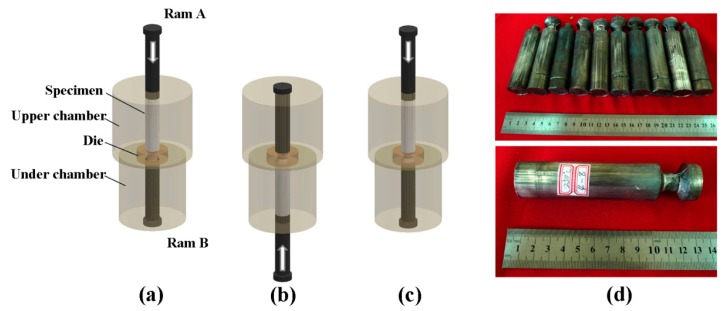
Schematic illustration of the cyclic extrusion and compression (CEC) procedure (**a**) initial state; (**b**) 1P; (**c**) 2P; (**d**) actual picture of the CECed ZK61 alloy rods.

The material used in the study was an as-extruded Mg-6.63 wt % Zn-0.56 wt % Zr alloy bar with a diameter of 30 mm. The specimens with 26 mm in diameter and 100 mm in length were machined out of the as-extruded bar and solution-treated at 400 °C for 48 h, and then lubricated with molybdenum disulfide (MoS_2_). After putting the specimen into the upper chamber of the CEC die, both the specimen and the CEC dies were heated to the designed temperature for about 30 min by a heating jacket that was coated on the die. The process that the specimen was extruded from one chamber to the other chamber was called one pass. In one pass, the specimen was extruded and compressed only once, and the compression occurred simultaneously with the extrusion under the two rams. The CEC process and die structure are illustrated in Figure 1. In this study, the specimens were subjected to a CEC process for different passes (2, 4, 8, 16 passes, which were denoted as 2, 4, 8, and 16 P, respectively), temperatures (200, 250, 300, 350 °C) and extrusion ratio 2. The CEC process was performed on a 4-column hydraulic press with a pressing velocity of about 5 mm/s. The interval time between the two passes was approximately 1 min. After CEC, additional annealing at 200 °C for 30 min (O-temper) was conducted on the CECed rods for eliminating the residual work hardening. Mechanical properties were examined by tension and compression at room temperature with a strain rate of 0.5 × 10^−3^ s^−1^. The dog-bone shaped specimen for tension was a gauge length of 15 mm and a diameter of 5 mm within the gauge section, and the cylindrical specimen for compression was 8 mm in diameter and 12 mm in length. Both were extracted from the annealed rod along the ED. Longitudinal sections of the annealed rod were sectioned for microstructure analyses by optical microscopy (OM) on OLYMPUS GX71, electron backscattering diffraction (EBSD) on JEOL 733 electron probe equipped with TSL OIM Analysis system. The grain size were studied by an ameliorated area-based averaging method [19]. The overall average area-based grain sizes, ${\bar{d}}_{A}$, were calculated using the area-based averaging method as:

(1) $${\bar{d}}_{A}={\left(\frac{4}{\pi }\frac{A}{N_{A}}\right)}^{-1/2}$$

where A represents the area of grains in given region; $N_{A}$ means the corresponding total amount of grains. As accepted, the relationship between the mean lineal intercept length, $\bar{L}$, and the average grain area, ${\bar{d}}_{A}$, for a circular grain section is:

(2) $$\bar{L}={\left(\frac{\pi }{4}\frac{A}{N_{A}}\right)}^{1/2}=\frac{\pi }{4}{\bar{d}}_{A}$$

A reasonable estimation of the spatial diameter, ${\bar{d}}_{V}$, based on the tetrakaidecahedron shape model:

(3) $${\bar{d}}_{V}=1.74\bar{L}=1.37{\bar{d}}_{A}$$

Thus, the average grain sizes calculated by area measurements were converted into the spatial diameters in the end, through multiplying the factor of 1.37 (1.74 × *π*/4). The “grain size” in the following text are all referring to the “average grain size”.

## 3. Results and Discussion

**Figure 2:**
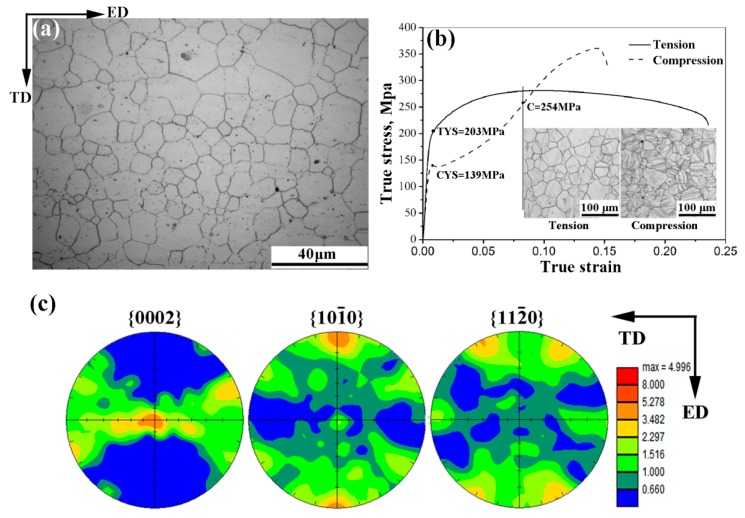
Microstructure and Mechanical behavior for as-extruded ZK61 alloy rods: (**a**) microstructure of as-extruded ZK61 alloy rods solution-treated at 400 °C for 48 h; (**b**) room-temperature true stress-strain curves in tension and compression; and, (**c**) {0002}, {10-10} and {11-20} pole figures.

Figure 2 displays the optical microstructure of the as-extruded ZK61 alloy rod solution-treated at 400 °C for 48 h in the ED-TD (transverse direction) plane. The initial material contained a nearly uniform recrystallized structure with an average grain size of about 28.9 μm, and exhibited an intense fiber texture with {0002} basal plane parallel to the ED as a result of the extrusion history of the rod. The true strain-stress curves in Figure 2b show two conspicuous curve shape: power-law curve in tension and concave-down curve in compression, indicating obvious tension/compression asymmetry along the ED. It is clear that the ratio of the yield strengthen in tension to the yield strengthen in compression, commonly denoted as TYS/CYS, is 1.46 here. Our previous studies on the extruded magnesium alloy rods showed that the reason caused this obvious tension/compression asymmetry was usually related to the contribution of {10-12} tension twinning to deformation at different stages. Chen et al. [20] suggested that slip-dominated deformation almost dominated the most part of strain hardening in tension, but twinning deformation dominated the early stage of strain hardening in compression and slip-dominated deformation began to dominate strain hardening only when the twinning hardening was completely saturated. The inflection point C of the concave compression curve is the saturation point of the twinning hardening. When comparing the longitudinal cross-section metallographic diagram of the tension and compression test specimen at the C-point strain in Figure 2b, it can be seen that the tension twinning in the compression specimen is almost saturated but almost nonexistent in tension specimen, which is consistent with the previous results. Therefore, it can be said that tension twinning plays an important role in the tensile and compressive mechanical properties of magnesium alloy. The occurrence of twinning is related to the preferred orientation of grains and the direction of applied load. The preferred orientation of grains determines the texture type. Many scholars have shown that the tensile and compressive properties of magnesium alloys can be affected to a great extent by microstructure refinement and texture evolution [21,22,23,24,25,26].

**Figure 3:**
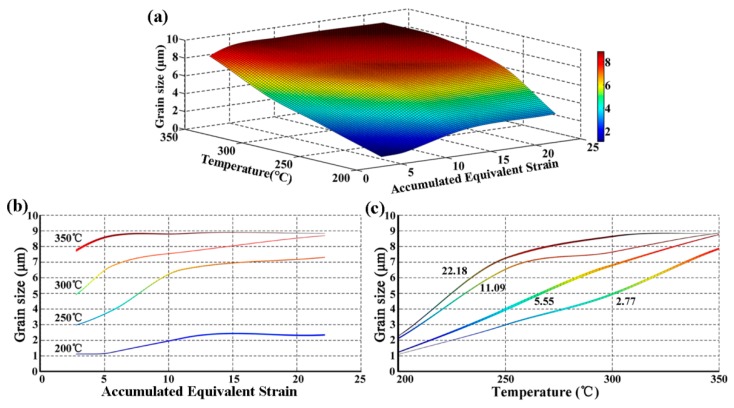
Three dimensional grain size distributions of CECed ZK61 alloy rods at different temperature and accumulated equivalent strains. (**a**) Three-dimensional (3D) diagram of grain sizes; (**b**) grain size curve of same temperature and different accumulated strains; and (**c**) grain size curve of same accumulated strains and different temperature.

In this study, the correlation between the accumulated equivalent strain and the grain size obtained by CEC at different temperature and passes is given in Table 1. The accumulated equivalent strain can be calculated according to the following equation [27]:

(4) $$\varepsilon =4nln\left(\frac{D}{d}\right)$$

where *n* is the number of CEC pass, *D* is the diameter of the upper chamber and the under chamber, and *d* is the channel diameter of the die. Figure 3a displays the three dimensional diagram of grain size distributions of CECed ZK61 alloy rods at different temperatures and accumulated equivalent strains. It can be seen that the lower temperature is more beneficial to grain refinement than accumulated equivalent strain. Figure 3b shows that the grain size slightly grows up with the increase of accumulative deformation at same temperature in detail. When the accumulated equivalent strains increase from 2.77 to 22.18, the grain size of the magnesium alloy increases from 7.81 to 8.85 μm at 350 °C and 1.1 to 3.2 μm at 200 °C, while the grain size increases from 4.89 to 8.68 μm at 300 °C and 3.0 to 7.29 μm at 250 °C, respectively. The main reason for the growth of grains is that most of the plastic deformation energy is converted into deformation heat during CEC. More specifically, it is the considerably short interval time between two passes that retards the dissipation of heat energy that results in the temperature increase in specimens and the consequent grain growth. Figure 3c shows that the grain size of the magnesium alloy decreases with the decreasing of the temperature at same accumulated equivalent strains in detail. When the temperature drops from 350 °C to 200 °C, the grain sizes sharply decrease from 7.81 to 1.1 μm at the cumulative deformation of 2.77 and from 8.85 to 3.2 μm at the cumulative deformation of 22.18, respectively. Under the same strain rate, the dynamic recrystallization (DRX) depends mainly on the temperature. In general, although DRX completes more fully and the microstructure is more uniform at the higher deformation temperature, the grain boundary diffusion and migration are also beneficial to grain growth, which leads to grain coarsening.

**Figure 4:**
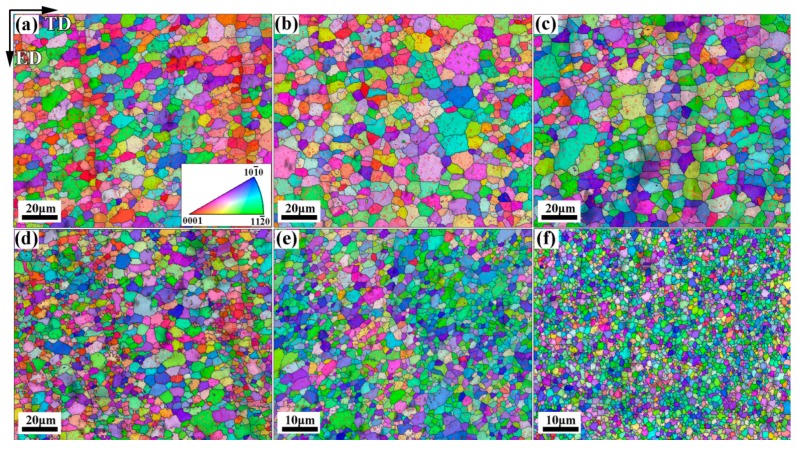
Microstructure of CECed ZK61 alloys rods at different passes and temperature: (**a**) 350 °C 2P; (**b**) 350 °C 4P; (**c**) 350 °C 16P; (**d**) 300 °C 2P; (**e**) 250 °C 2P; and, (**f**) 200 °C 2P.

**Figure 5:**
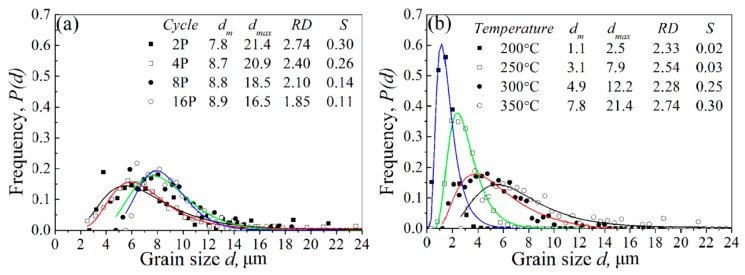
Grain size distribution of CECed ZK61 alloy rods at (**a**) 350 °C and (**b**) 2P.

The observed microstructures of CECed ZK61 alloy rods at different passes and temperatures are shown in Figure 4. The corresponding grain size distributions (*P*(*d*), *d* means the area-based grain size) presented in Figure 5 are fitted by the log-normal distribution function [26]. Regarding the microstructural heterogeneity, the standard deviation of the variable in ln*d*, *S*, and the relative diameter range, *RD* = *d*_max_/*d*_m_ [28], are used to characterizing grain size dispersions. Figure 5a shows that the increasing of CEC passes is beneficial to the homogenization of the grains at the same temperature. Figure 5b shows the lower temperature can lead to more significant grain refinement and homogenization by CEC than the passes. As shown in Figure 5a, recrystallized structure with average grain size of 7.81 μm was formed after the 2nd pass at 350 °C. The presence of band-like structure consisted of smaller grains of 2.2 μm due to the localized SPD leads to a broad grain size dispersion as characterized by *S* of 0.3 and *RD* of 2.74, indicating a beneficial effect of microstructure heterogeneity of grains. As the number of passes increases, the grains grow slightly and become more uniform. After sixteen passes, the microstructure finally evolved into the structure with an average grain size of 8.85 μm, whose *S* and *RD* are merely 0.11 and 1.85, respectively. When compared to Figure 5a, Figure 5b shows that the grains can be refined to 1.1 μm only by two passes at 250 °C, whose *S* and *RD* are 0.03 and 2.54, respectively. With the decrease of temperature, the grains can be further refined and the microstructure heterogeneity is further improved under the same pass. The grains are refined to 1.1 μm at 200 °C, whose *S* and *RD* are 0.02 and 2.33, respectively. Therefore, low temperature is more favorable for the grain refinement of magnesium alloy.

**Figure 6:**
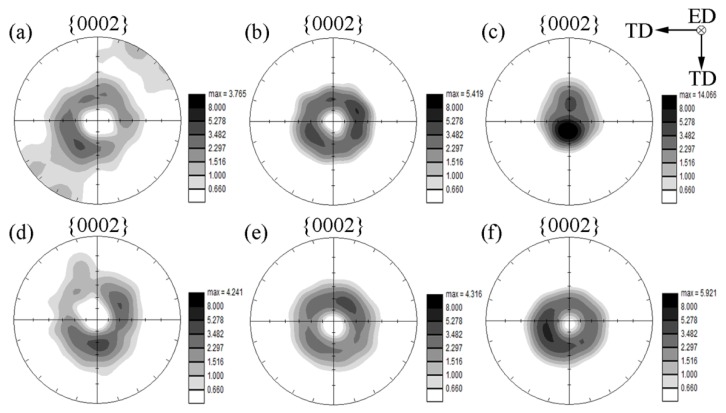
{0002} pole figures of CECed ZK61 alloy rods: (**a**) 350 °C 2P; (**b**) 350 °C 4P; (**c**) 350 °C 16P; (**d**) 300 °C 2Ps; (**e**) 250 °C 2P; and, (**f**) 200 °C 2P.

**Figure 7:**
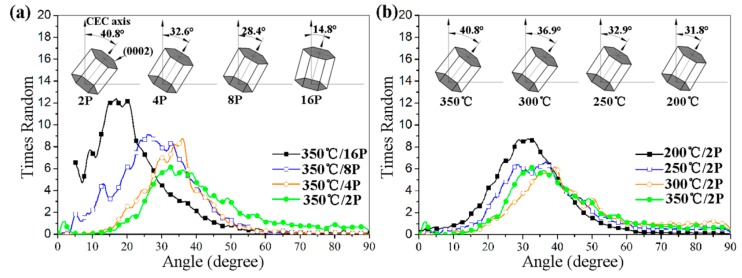
{0002} pole plot of CECed ZK61 alloy at (**a**) 350 °C and (**b**) 2P.

The {0002} pole figures of CECed ZK61 alloy rods at different passes and temperatures are shown in Figure 6. Apparently, the grains of the ZK61 alloy presented an obvious preferred orientation after CEC. However, this preferred orientation is different from the typical fiber texture by the conventional extrusion of magnesium alloy. After CEC, the c-axes of the grains are inclined at an angle of 15° to 40° with the ED of the magnesium alloy, which can be approximated as (10-13)<30-32> texture [29]. With the increase of the pass or the decrease of temperature, the maximal density of the texture increases gradually, and the angle between the c-axis of the grain and the ED of the magnesium alloy, which is denoted as *θ*, decreases slightly. The corresponding (0002) pole plot of CECed ZK61 alloy is presented in Figure 7. Hypothetically a horizontal line descends from the top of the curve and produces two cross-points on the curve. When the area between the curve, the axis x and the crosspoints is 60% of the total area, the average of the two crosspoints is seen as *θ*. Figure 7a shows *θ* decreases with the increase of CEC passes at same temperature. The *θ* is about 40.8° after the 2nd pass at 350 °C. With the increase of CEC pass, the c-axes of the grains gradually turn to the extrusion axis. When the number of passes reaches 16, the *θ* becomes 14.8°. Figure 7b shows *θ* slightly narrows with the decrease of temperature at two passes. When the temperature drops from 350 °C to 200 °C, *θ* decreases from 40.8° down to 31.8°. It can be seen that both the temperature and the passes in CEC processing determine *θ*.

**Figure 8:**
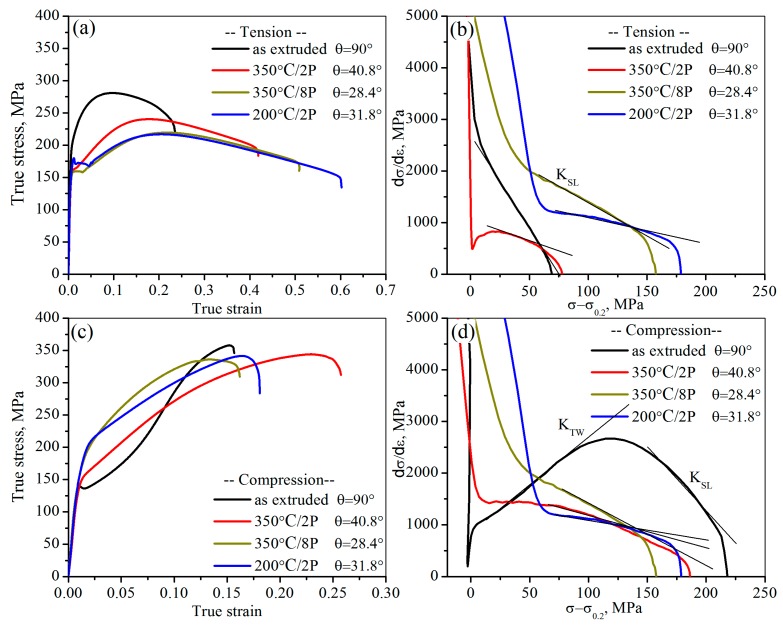
Room-temperature true stress-true strain curves and hardening curve for CECed ZK61 alloy rods in tension (**a**,**b**) and in compression (**c**,**d**).

In order to ascertain the influence of grain sizes and *θ* on the mechanical responds and predominant deformation mechanisms during deformation in tension and compression, the room-temperature true stress-true strain curves and hardening curves for CECed ZK61 alloy rods in tension and compression are analyzed in Figure 8. As shown in Figure 8a,c, the yield strength (0.2% proof stress) decreases monotonically when comparing to the initial as-extruded ZK61 alloy rods in tension but increases in compression despite of grain refinement. The curves in both tension and compression change significantly when comparing to the initial after CEC. The tensile curve exhibits a pronounced yield platform, which is completely different from the initial tensile curve of, a typical power-law curve. The corresponding yield strength decreases from 203.0 MPa of the initial to 150.9 MPa after 350 °C/8P. Although the yield strength is slightly improved with grain refinement after 200 °C/2P, the tension yield strength is still much lower than that of the initial. On the contrary, the compression curves evolve into a power-law curve similar to the tension curve of the as-extruded ZK61 from the initial concave-down curve, which results in a higher compression yield strength than that of the initial after CEC. After 350 °C/4P and 350 °C/8P the average grain size basically remains at about 8.8 μm, while the corresponding compression yield strengths are 164.2 and 178.9 MPa, respectively. The difference of the compression yield is mainly attributed to the decrease of the *θ* to enhance the compression yield strength of ZK61 alloy. The variation of *θ* will lead to the change of the grains orientation which in turn results in the alteration of the deformation mechanism in tension and compression. The strain hardening behaviors of the specimens in tension and compression are analyzed in Figure 8b,d. According to previous literature [30], the positive slope *K*_tw_ and the negative slope *K*_SL_ of the strain hardening curve were attributed to the twinning-induced deformation and the slip-dominated deformation, respectively. A higher positive slope, *K*_tw_, which is correlated with a greater contribution of twining to plastic deformation, while a steeper negative (higher in absolute value) slope *K*_SL_ with a greater contribution of non-basal slip. As for the tension, the slope *K*_SL_ of the initial as-extruded ZK61 is −36.2, indicating that the basal slip almost dominated the entire tension deformation. After 350 °C/8P and 200 °C/2P, the slope *K*_SL_ gradually becomes −12.3 and −5.3, respectively, which means that the greater no-basal slips will contribute to tension deformation. The compression strain hardening curve changes significantly after CEC. The slope *K*_tw_ of the initial as-extruded ZK61 alloy rod almost disappear and completely evolve into a shape similar to the tension hardening curve after 350 °C/8P and 200 °C/2P. Their slope *K*_SL_ are −12.8 and −4.1, which means that both the basal slip and no-basal slip will contribute to compression deformation like the tension deformation of the CECed ZK61 alloy rods.

**Figure 9:**
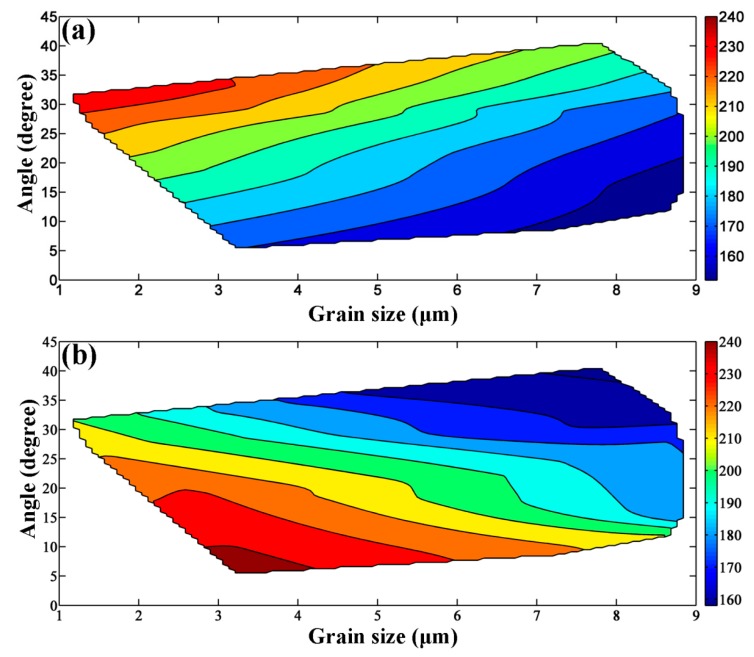
Room-temperature yield strength contour map of the CECed ZK61 alloy rods as a function of the grain size and *θ*: (**a**) tension; (**b**) compression.

**Figure 10:**
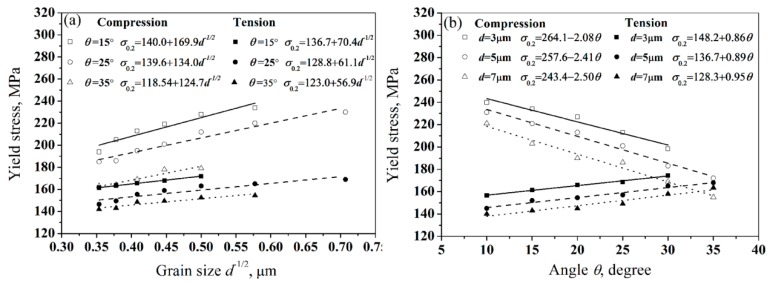
Variations of strengths of CECed ZK61 alloy rods as a function of (**a**) grain size or (**b**) the angle, *θ*, between the c-axis of grains and the ED in tension and compression.

In order to discuss the influence of grain sizes and *θ* on the mechanical properties of ZK61 alloys in detail, the data of the grain sizes, *θ*, the yield strength of the CECed ZK61 alloy rods in tension and compression at the different temperatures and passes are summarized in Table 2. The yield strength contour map of the CECed ZK61 alloy rods as a function of the grain size and *θ* by Matlab surface fitting is shown in Figure 9. It can be seen that both tension yield strength and compression yield strength increase with grain refinement, while the change trend of them is opposite with the increasing of *θ*. In order to further analyze the effect of grain size on the yield strength of ZK61 alloy under the condition of fixed texture, we fixed the angle *θ* to measure the yield strength of different grain sizes in Figure 9. The measured yield strength is plotted as a function of the inverse square root of the corresponding grain size in Figure 10a. The linear regression of measured data for each angle *θ* clearly demonstrates that H-P relationship can be established on the fixed angle *θ*. Different intercepts (*σ*_0_) and slope (*k*_θ_) that were obtained for three different angle *θ* (15°, 25° and 35°) are summarized in the legend of Figure 10a. It is shown that the *k*_θ_ and *σ*_0_ increase with the decrease of *θ* in tension or compression, which indicates the ZK61 alloy with lower *θ* contains a stronger grain size sensitivity. When comparing the tension and compression yield strength, compression is more sensitive to the grain size under the same texture conditions. In summary, the result shows that both the tension yield strength and the compression yield strength of ZK61 alloy are fully consistent with the Hall-Petch relationship in the condition of certain texture. In the same way, in order to analyze the influence of the angle *θ* on the yield strength of magnesium alloy under the same grain size, we fixed the grain size to measure the yield strength of different angle *θ* in Figure 9. The measured yield strength is plotted as a function of the corresponding angle *θ* in Figure 10b. Similarly, the linear regression of measured data for each angle *θ* also demonstrates a linear relationship on the same grain size *d*, but the change trends of the tension yield strength and the compression yield strength are completely opposite while *θ* alters. Different intercepts (*σ*_0_) and slope (*k*_θ_) obtained for three different grain sizes (3, 5 and 7 μm) are summarized in the legend of Figure 10b. The absolute value of *k*_θ_ increases with the increase of grain size *d* in tension or compression, which indicates the ZK61 alloy with larger grain size contains the stronger texture sensitivity. When comparing the tension and compression yield strength, compression is more sensitive to texture under the same grain size.

The deformation model of magnesium alloy at ambient temperature includes the following three slip systems and one twinning system: (i) basal slip {0001}<11-20>; (ii) prismatic slip {10-10}<11-20>; (iii) pyramidal slip {11-22}<11-23> and {10-11}<11-20>; and, as well as (iv) twinning slip {10-12}<10-11> [4]. Only when the shear stress along the slip direction on the slip plane is greater than the critical resolved shear stress (CRSS) of the slip system, the slip system can be activated. Referring to the CRSS for basal, prismatic, and pyramid slip as well as extrusion twin at ambient temperature obtained from the previous literature [31,32,33,34], we assume they would agree with the following ratios:

$$\tau_{\mathrm{CRSS}}^{\mathrm{basal}}:\tau_{\mathrm{CRSS}}^{\mathrm{Prismatic}}:\tau_{\mathrm{CRSS}}^{\mathrm{Pyramidal}}:\tau_{\mathrm{CRSS}}^{\mathrm{twin}}=1:2:4:0.7$$

**Figure 11:**
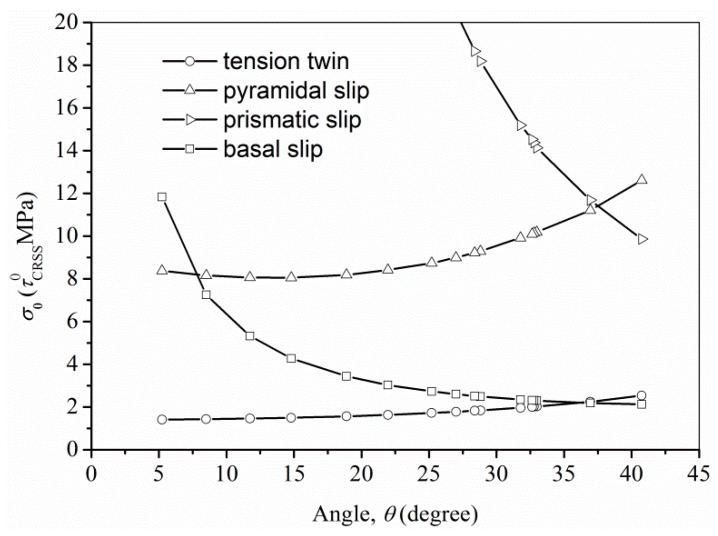
Calculated friction stress, *σ*_0_, for different deformation modes as a function of the angle, *θ*, between the c-axes of the grains and the ED based on Table 4.

Therefore, the yield strength is directly related to the activation of a particular deformation mechanism and is significantly affected by the angular relationship between the mechanical loading direction and its crystallographic texture in wrought magnesium alloys [35]. In the Hall-Petch relationshi, $\sigma_{0}$ is the friction stress for dislocation movement, at which material yields if the grain-boundary effects are not considered. According to Armstrong et al. [36], $\sigma_{0}$ can be written as:

(5) $$\sigma_{0}=\tau_{\mathrm{CRSS}}/m$$

where $\tau_{\mathrm{CRSS}}$ is the CRSS of a specific deformation mode and *m* is a suitable average Schmid factor for the polycrystal. Assuming that the c-axes of all the grains in rod have a certain angle *θ* with the ED after CECed, the Schmid factors of the different slip systems for the various angle *θ* can be calculated (see Table 3). It is worth noting that the “ideal” Schmid factor is the average of the Schmid factors along six different slip directions in HCP, assuming the angle, *θ*, between the c-axes of the grains and the ED is constant. In this study, we assume that the CRSS of ZK61 alloy for the basal slip is the constant $\tau_{\mathrm{CRSS}}^{0}$. The friction stress, $\sigma_{0},$ is calculated for different deformation modes for the different angle *θ*, and the results are presented in Table 4. After CEC at different temperatures and different passes, the C-axes of the grains in the magnesium alloy rods vary only at an angle of 5° to 40° to the ED. In order to more clearly show the variation of $\sigma_{0}$ on the different slip system of the magnesium alloy with the angle *θ*, the fitting curves of the calculated $\sigma_{0}$ as a function of the angle *θ* are shown in Figure 11. The lowest curve under the same *θ* represent the dominant deformation mechanism at yielding. The fitting curve indicates that from $\theta =5^{{}^{\circ}}$ to 36°, tension twinning is dominant and the basal slip will also contribute mostly at yielding, while the contribution from the basal slip obviously decreases with the decreasing angle *θ*. However, it is worth noting that the tension twinning hardly occurs when the grain is subjected to compression loading along the c-axis due to the well-known twinning polarity, which results in different deformation mode in tension and compression yielding. The prismatic slip might only occur when the angle *θ* is less than 7° and the pyramid slip is impossible to occur. Therefore, the tension yield strength of the CECed ZK61 alloy rods is determined by both the basal slip and the tension twinning slip during the tension deformation at room temperature, while the compression yield strength is determined only by the basal slip during the compression deformation. Moreover, the contribution of the basal slip becomes smaller and smaller with the decrease of the angle *θ* in tension, while the contribution of the tension twinning becomes larger and larger. The results are in agreement with the increasing basal-slip contribution as analyzed from the negative slop in compression in Figure 10b.

## 4. Conclusions

The ZK61 alloy rods with different grain sizes and crystallographic texture were successfully fabricated by cyclic extrusion and compression (CEC). The contour map of room-temperature tension and compression yield strength in ZK61 alloy rods as a function of grain size and the texture was obtained and analyzed, which made it possible to distinguish the strengthening mechanism of texture and grain size on the tension & compression yield strength. This can provide an available reference to processing and designing high-performance wrought Mg alloy rods that were suitable for industrial fabrication. The conclusions are as follows:

(1) The CEC processing is an efficient grain refinement way for the ZK61 alloy. Low temperature is more favorable for the grain refinement of ZK61 alloy than the increase of passes.
(2) The contour map of room-temperature tension & compression yield strength in ZK61 alloy rods as a function of grain size and the texture was constructed by means of quantifying the texture by the angle, *θ*, between the c-axes of the grains and the ED, which made it possible to distinguish the strengthening mechanism of texture and grain size on the tension & compression yield strength.
(3) The results shows that whether the tension yield strength or the compression yield strength of ZK61 alloy is fully consistent with the Hall-Petch relationship in certain texture, but the change trends of the tension yield strength and the compression yield strength are completely opposite with the increase of *θ* under the same grain size.
(4) The tension deformation of the CECed ZK61 alloy rods is determined by both the basal slip and the tension twinning at room temperature, while the compression deformation is determined only by the basal slip.

## Figures and Tables

**Table 1:** Summarized parameters of CEC for as-extruded ZK61 alloy rods.

Temperature (°C)	Extrusion Ratio	CEC Pass	Accumulated Equivalent Strain	Grain Size (μm)
350	2	2	2.77	7.81
		4	5.55	8.73
		8	11.09	8.84
		16	22.18	8.85
300	2	2	2.77	4.89
		4	5.55	6.79
		8	11.09	7.62
		16	22.18	8.68
250	2	2	2.77	3.0
		4	5.55	3.87
		8	11.09	6.51
		16	22.18	7.29
200	2	2	2.77	1.1
		4	5.55	1.3
		8	11.09	2.7
		16	22.18	3.2

**Table 2:** Summarized mechanical properties of the CECed ZK61 alloy rods.

Temperature (°C)	Accumulated Strains	Grain Size (μm)	*θ* (°)	YS (MPa)	
				Ten.	Com.
350	2.77	7.81	40.75	162.5 ± 2.1	157.8 ± 3.1
	5.55	8.73	32.63	160.5 ± 1.9	164.2 ± 4.3
	11.09	8.84	28.38	150.9 ± 4.3	178.9 ± 2.8
	22.18	8.85	14.8	135.9 ± 3.1	185.1 ± 3.6
300	2.77	4.89	36.94	165.5 ± 2.4	165.9 ± 2.1
	5.55	6.79	32.77	160.0 ± 2.1	170.6 ± 2.8
	11.09	7.62	25.19	146.8 ± 4.1	186.6 ± 4.2
	22.18	8.68	11.74	135.5 ± 2.7	211.8 ± 5.1
250	2.77	3.0	32.98	178.8 ± 3.3	189.7 ± 3.5
	5.55	3.87	28.81	168.7 ± 2.6	195.5 ± 2.2
	11.09	6.51	21.96	147.9 ± 1.5	200.9 ± 4.1
	22.18	7.29	8.5	143.8 ± 2.1	222.8 ± 5.0
200	2.77	1.1	31.78	185.7 ± 2.4	210.0 ± 2.6
	5.55	1.3	27.0	180.8 ± 2.2	217.4 ± 3.2
	11.09	2.7	18.89	165.5 ± 5.1	231.7 ± 2.1
	22.18	3.2	5.23	150.6 ± 4.8	246.1 ± 2.7

**Table 3:** Ideal Schmid factors, *m*, for basal slip, prismatic slip, pyramid slip and extension twin as a function of the angle, *θ*, between the c-axis of the grains and the extrusion direction (ED).

Angle, *θ* (°)	Basal Slip {0001}<11-20>	Prismatic Slip {10-10}<11-20>	Pyramid Slip {10-12}<11-20>	Extension Twin {10-12}<10-11>	
				Ten.	Com.
40.75	0.4717	0.2029	0.3172	0.2763	0
36.94	0.4577	0.1712	0.3571	0.3121	0
32.98	0.4352	0.1415	0.3927	0.3464	0
32.77	0.4338	0.1392	0.3944	0.3481	0
32.63	0.4324	0.1377	0.3962	0.3498	0
31.78	0.4266	0.1317	0.4034	0.3565	0
28.81	0.4019	0.11	0.4305	0.3808	0
28.38	0.3983	0.1072	0.4339	0.3839	0
27.0	0.385	0.0977	0.445	0.3945	0
25.19	0.3665	0.0858	0.4579	0.407	0
21.96	0.3302	0.0658	0.4754	0.4292	0
18.89	0.2911	0.0495	0.4889	0.4477	0
14.8	0.2342	0.0307	0.4969	0.4681	0
11.74	0.1879	0.0193	0.4966	0.4803	0
8.5	0.1379	0.0102	0.4903	0.4898	0
5.23	0.0845	0.0038	0.4778	0.497	0

The ideal Schmid factor is the average of the Schmid factors along six different slip directions in HCP assuming the angle, *θ*, between the c-axes of the grains and the ED is constant.

**Table 4:** Calculated friction stress, *σ*_0_, for different deformation modes as a function of the angle, *θ*, between the c-axes of the grains and the ED based on Table 3.

Angle, *θ* (°)	Calculated Friction Stress, *σ*_0_ ($\tau_{\mathrm{CRSS}}^{0}\cdot \mathrm{MPa}$)				
	Basal Slip {0001}<11-20>	Prismatic Slip {10-10}<11-20>	Pyramid Slip {10-12}<11-20>	Extension Twin {10-12}<10-11>	
				Ten.	Com.
40.75	2.11999	9.85707	12.61034	2.53348	0
36.94	2.18484	11.68224	11.20134	2.24287	0
32.98	2.29779	14.13428	10.18589	2.02079	0
32.77	2.30521	14.36782	10.14199	2.01092	0
32.63	2.31267	14.52433	10.09591	2.00114	0
31.78	2.34412	15.18603	9.91572	1.96353	0
28.81	2.48818	18.18182	9.29152	1.83824	0
28.38	2.51067	18.65672	9.21871	1.82339	0
27.0	2.5974	20.47083	8.98876	1.7744	0
25.19	2.72851	23.31002	8.73553	1.7199	0
21.96	3.02847	30.39514	8.41397	1.63094	0
18.89	3.43525	40.40404	8.18163	1.56355	0
14.8	4.26985	65.14658	8.04991	1.49541	0
11.74	5.32198	103.62694	8.05477	1.45742	0
8.5	7.25163	196.07843	8.15827	1.42915	0
5.23	11.83432	526.31579	8.3717	1.40845	0
